# Anogenital Distance and Condition as Predictors of Litter Sex Ratio in Two Mouse Species: A Study of the House Mouse (*Mus musculus*) and Mound-Building Mouse (*Mus spicilegus*)

**DOI:** 10.1371/journal.pone.0074066

**Published:** 2013-09-19

**Authors:** Péter Szenczi, Oxána Bánszegi, Zita Groó, Vilmos Altbäcker

**Affiliations:** 1 Department of Ethology, Eötvös Loránd University, Göd, Hungary; 2 Institute for Soil Sciences and Agricultural Chemistry, Centre for Agricultural Research, Hungarian Academy of Sciences, Budapest, Hungary; University of Turku, Finland

## Abstract

The Trivers – Willard hypothesis (1973) suggests that the maternal condition may affect the female's litter size and sex ratio. Since then other factors had been found. Previous findings revealed in the case of some mammalian species, that females with larger anogenital distance have smaller litters, while the sex ratio is male-biased. That has only been demonstrated in laboratory animals, while the genetic diversity of a wild population could mask the phenomenon seen in laboratory colonies. We examined the connection between morphological traits (weight and anogenital distance) and the reproductive capacity of two wild mice species, the house mouse and the mound-building mice. We showed in both species that anogenital distance and body weight correlated positively in pre-pubertal females, but not in adults. Neither the house mouse nor the mound-building mouse mothers' weight had effect on their litter's size and sex ratio. Otherwise connection was found between the mothers' anogenital distance and their litters' sex ratio in both species. The results revealed that females with larger anogenital distance delivered male biased litter in both species. The bias occurred as while the number of female pups remained the same; mothers with large anogenital distance delivered more male pups compared to the mothers with small anogenital distance. We concluded that a female's prenatal life affects her reproductive success more than previously anticipated.

## Introduction

Several previous studies have shown that in a number of mammalian species the sex ratio of offsprings in utero or at birth may differ from 50:50. Almost 40 years ago Trivers and Willard suggested that natural selection favors maternal control of offspring sex ratio: the decline of maternal condition may produce lower ratio of males [1]. Since then, it was found that this hypothesis applies to species in which the litter size is one. In the case of multiparous species, the published reports are controversial [2]–[4]. In general, a females' social status affects its litter's size and sex ratio, where high ranking mothers gave birth to larger and male biased litters [5], [6]. High rank generally means better quality or condition, larger body size or weight, and better access to resources. Food restriction or a low fat diet during pregnancy leads to female biased litters in rodent females [6]–[11]. However, a number of studies did not support the Trivers – Willard theory. Maternal condition did not affect the sex ratio in wild-caught mice [12] or alpine marmot [13]. Wild boar mother quality (size and weight) affected only the size of the litter, but not the sex ratio [14].

Clark et al. [15] suggests another factor, the females' prior intrauterine position (IUP), which can influence her litters' sex ratio. In several polytocous mammals, the sexual differentiation of a female fetus is known to be affected by the testosterone produced by adjacent males [16], [17]. The observed effect of testosterone reaching the fetuses is shown to be dose dependent. Female mice having 2 adjacent male littermates (2M females) become less feminine in morphology, physiology and behavior than those bearing one or no adjacent males (1M and 0M females) [18], [19].

Anogenital distance (AGD, the distance between the anus and the genitalia) is frequently used as a biomarker of natural variation in prenatal androgenization, and it shows gender differences already at birth. Additionally, it reflects the IUP, as 2M females have longer AGD than 0M females, while 1M females are intermediate in many species both at birth and in adulhood (mice [20]–[24], rats [16], [25], [26], Mongolian gerbils [27] and rabbits [28].

Prenatal androgenization has long term effects on the physiology of the animals as well as their behavior. Female mice with 0M intrauterine position show vaginal openings at an earlier age [21], shorter estrous cycles [29] and are more sexually attractive and arousing to males [17], [24], show less aggressive behavior [30] and give birth to more litters [31] during their lifetime than 2M females. 2M female mice and gerbils have higher testosterone concentration in their blood [32], [33] and it is known that the maternal hormonal status can affect the sex ratio of the litter via embryo mortality and resorption [34]. Both 2M female Mongolian gerbils and house mice produce male biased litters [15], [35], [36]. Domesticated and wild type rabbit females with larger AGD have smaller litters and its sex ratio is male biased [37], [38].

The published literature is controversial about the reproductive performance of house mice. These contradictions might arise because researchers use different laboratory mice strains, and genetic factors cause litter size and sex ratio variations. Some inbred strains produce male-, while others produce female-biased litters and the average litter sizes also differs among them [39], [40]. Litter sizes of CD-1 mice increase with maternal weight [9], but maternal weight at mating had no effect on litter size nor sex ratio in wild-caught house mice [12].

In mice, the fetuses' IUP is reflected in their AGD [22], [23] and the female's IUP correlates with their litter's sex ratio [35]. However, the second connection was examined only in laboratory stock. It raises the question, whether this phenomenon can only be detected in genetically homogeneous populations, or if it is also important in wild populations. The genetic diversity of a wild population could theoretically mask this phenomenon seen in laboratory colonies; hence it would have no real importance on population structure and dynamics. The aim of this study was to investigate whether the weight or the AGD is an indicator of the reproductive capacity in wild mice. To answer this question specimens of two wild mice species native to Hungary, the house mouse (*Mus musculus musculus*, HM hereafter) and the mound-building mouse (*Mus spicilegus*, MBM hereafter) differing in their reproductive traits were captured and bred for the study. Most studies examine the HM, so our results are comparable with the previous ones. Using AGD as a biomarker to measure reproductive consequences of intrauterine hormone exposure is simple and non-invasive method that can be used on wild animals [41]. This technique allows field research of the impact of intrauterine position possible.

## Materials and Methods

### Ethics statement

The procedure used in this study was approved by the Ethical Committee for Animal Experiments at Eötvös Loránd University, and followed the rules detailed in the guidelines of the American Society of Mammalogists [42] and the European Communities Council Directive of 24 November 1986 (86/609/EEC).

### Materials and methods

The experimental animals were 1st-2nd generation laboratory born descendants of wild caught animals. Founding populations were live-trapped in fields and farmhouses in three distant locations in Hungary. The MBM were collected from mounds found on fields, while HM were trapped at the vicinity of nearby farms.

The mice were kept at the Biological Station of Eötvös Loránd University in Göd, Hungary, under laboratory conditions in standard polycarbonate cages (35×20×15 cm), between 18–21°C with 12:12 h reverse L/D cycle, with red light between 08:00 and 20:00 hour. We used sawdust as bedding material (LIGNOCELL from J. Rettenmaier & Söhne GmbH, Rosenberg, Germany), hay of alfalfa were provided for nest material. The animals were offered food pellets (Ssniff S8106-SO11 Spezialdiäten GmbH, Soest, Germany) and water *ad libitum*. All animals were weaned at 21 days of age. Females were housed in same sex group with their sister until pairing, while males were housed individually.

Some authors suggest using normalized AGD (AGDI – anogenital distance divided by the animal's weight) [41] but previous findings on the connection between these two traits are controversial and may differ between species and age. To justify the usage of either variable, these parameters were measured on 100 pre-puberty animals (50 HM females, 50 MBM females) on day 21 postpartum when weaning and the same measurements were conducted on 100 adult animals (50 – 50 HM and MBM females) at the age of 120 days. At that age even the MBM with delayed maturity are considered adults [43], [44]. The animal's AGD was measured to the nearest 0.01 mm using a digital caliper under a binocular microscope, three measurements were conducted on each individual and the averaged values were used. Mice's weight was registered to the nearest 0.01 g using a digital scale.

To test how an individual's AGD reflects their reproductive capacity 32 female virgin HM and 44 virgin MBM were weighed and their AGD measured before pairing with randomly selected males. After pregnancy was evident, the males were removed from the female's cage. The size of the litter was determined the day after partition; and the sex ratio of the litter was determined on day 8 postpartum. Litters with mortalities before sexing were excluded from the experiment.

In the comparison of the litter's sex composition, females were divided into two equal groups based on their AGD size [45]. The median of the AGD was calculated and individuals with a value under the median were treated as small AGD females, individuals above were treated as large AGD females.

### Statistical analysis

Relation of key variables to certain dependent measures was analyzed by Pearson's rank-order correlation tests. Variables were tested for normal distribution with the Kolmogorov–Smirnov test. Litter size and sex ratio (as males/litter size) data were analyzed using Generalized Linear Model (GLM) with quasipoisson error distribution following Hardy [46].

Differences in the number of males and female pups were analyzed using GLM with quasipoisson error distribution. Species, AGD-type and mother's weight were part of the original model, but non-significant factors were removed after ANOVA model comparisons. Summary data are reported as mean ± *SE*, unless stated otherwise. Statistica 8.0 [47] and the R package ‘RCMDR’ [48] statistical softwares were used.

## Results

### Connection between AGD and weight in prepubertal and adult female mice

**Figure 1:**
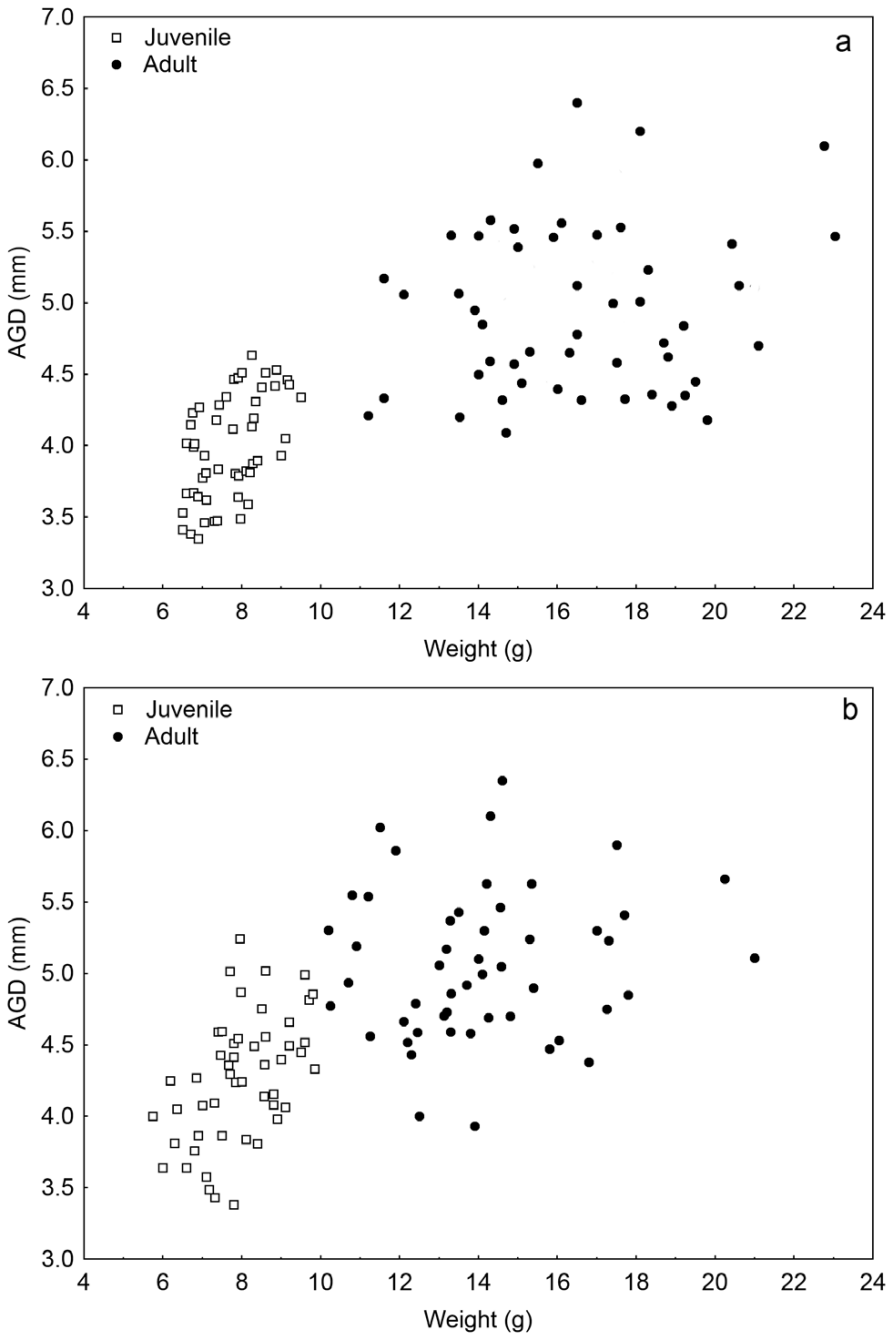
a. The relationship between anogenital distance (AGD) and weight in HM measured at day 21 and at day 120. Different age is indicated by the differently styled data points. Positive correlation was found in pre-pubertal mice, but it disappeared later on. **b. The relationship between anogenital distance (AGD) and weight in MBM measured at day 21 and at day 120.** Different age is indicated by the differently styled data points. Positive correlation was found in pre-pubertal mice, but it was not observed subsequently.

Data on the average weight and AGD of 21 and 120 days old HM and MBM females can be found in Table 1. Using Pearson's rank-order correlation test, anogenital distance and body weight showed positive correlation in pre-pubertal females in both species (HM: r = 0.54; n = 50; *p*<0.001; MBM: r = 0.49; n = 50; *p*<0.001), but this relation disappeared in adults (HM: r = 0.13; n = 50; *p* = 0.37; MBM: r = 0.10; n = 50; *p* = 0.47) (Figure 1.a.b). Since we did not find correlation between the body weight of adult females and the size of their AGD, normalization of AGD by weight was deemed unnecessary for the subsequent tests.

**Table 1:** The average weight (g ± *SE*) and AGD (mm ± *SE*) of 21 and 120 days old HM and MBM females.

	HM (n = 50)	MBM (n = 50)
Weight at day 21	7.71±0.12	7.97±0.15
AGD at day 21	3.98±0.05	4.27±0.06
Weight at day 120	16.54±0.39	14.08±0.34
AGD at day 120	4.95±0.08	5.06±0.07

### Effect of mothers' weight and AGD on litter characteristics

Data on the litters of HM and MBM females can be found in Table 2. Litter sizes of mice depended on the species of the mother but not on other measured variables (AGD: *t* = 1.277, *p* = 0.20, weight: *t* = 1.029; *p* = 0.30, species: *t* = 2.168, *p*<0.05; residual deviance (*RD*) = 18.66 on 70 *df*, dispersion parameter (*DP*) = 0.273).

**Table 2:** The average litter sizes (# ± *SE*), number of male and female pups (# ± *SE*) and sex ratios (% ± *SE*) of HM and MBM mothers.

	HM (n = 32)	MBM (n = 44)
Litter size	6.13±0.25	6.93±0.19
Number of males	2.96±0.22	3.47±0.18
Number of females	3.12±0.26	3.45±0.23
Sex ratio	0.49±0.03	0.51±0.02

**Figure 2:**
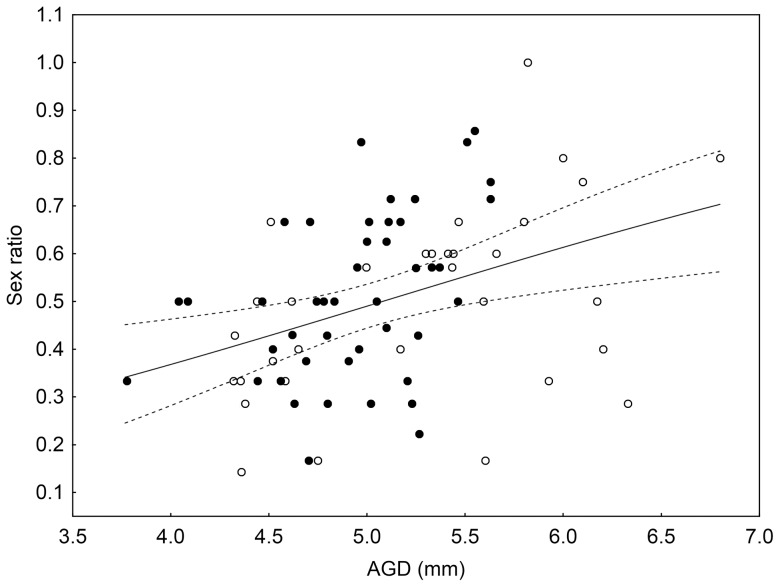
Correlation between the AGD of females' and the sex ratio of their litters. Each data point represents the AGD of a female and the sex ratio of its first litter. Empty circles represent HM females, sold circles represent MBM females. Higher sex ratio values mean male biased litters. Solid and dashed lines show model prediction with 50 points in the range with 95% confidence interval.

GLM taking males/litter size as dependent variable, species, mother's weight and AGD as independent variables revealed that only the mother's AGD had significant effect on the litters' sex ratio (AGD: *t* = 4.402; *p*<0.001; weight: *t* = 0.999; *p* = 0.32; species: *t* = 1.05, *p*<0.92; *RD* = 4.13 on 70 *df*, *DP* = 0.054). Larger AGD led to male biased sex ratio in the litters of both species. (Figure 2).

Bias in sex ratio can occur by changing the number of the members of one sex or both. For detailed examination of the sex composition of litters, mothers were divided into two groups – separately in both species – following Drickamer [49]: those with an AGD below median (small AGD, n = 16 and n = 22, HM and MBM respectively) and those with an AGD above the median value (large AGD, n = 16 and n = 22, HM and MBM respectively). The result of grouping the females was identical whether we used AGD or AGDI.

**Figure 3:**
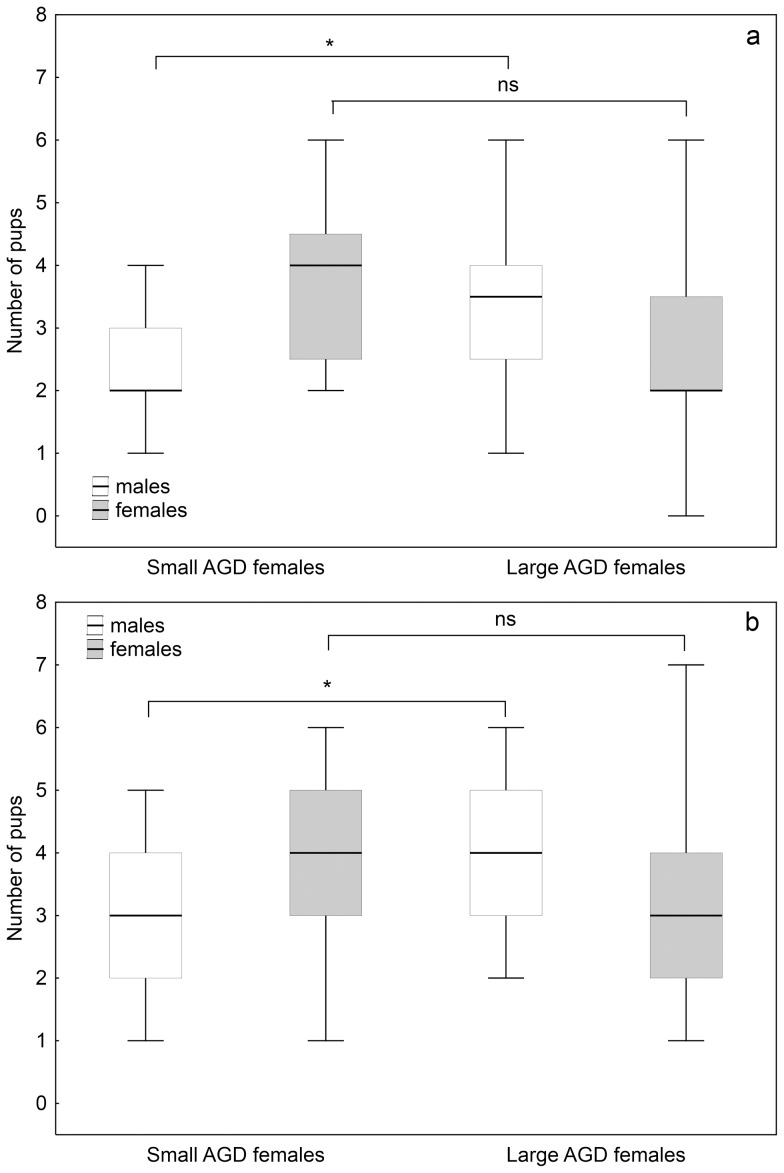
a. Number of male and female pups in the litters of small and large AGD HM. The data are presented as median (thick line), upper and lower quartiles (boxes) and minimum–maximum (whiskers). Mothers' AGD-type had significant effect only on the number of male pups. Details of statistical analysis can be found in the text. Asterisk mark significant differences. **b. Number of male and female pups in the litters of small and large AGD MBM.** The data are presented as median (thick line), upper and lower quartiles (boxes) and minimum–maximum (whiskers). Mothers' AGD-type had significant effect only on the number of male pups. Details of statistical analysis can be found in the text. Asterisk mark significant differences.

The original model consisted species, AGD-type and mother's weight as factors. Because the species effect on the number of males was slightly significant (*t* = 1.973, *p* = 0.052, *RD* = 28.92 on 70 *df*, *DP* = 0.392) we decided to analyze HM and MBM separately. Mother's weight as non-significant factor was removed from all models. Number of male pups was higher, while number of female pups was lower in the litters of large AGD mothers in both species, but only in the case of male pups was the AGD-type effect significant (HM males: *t* = 2.266, *p*<0.05, *RD* = 14.15 on 30 *df*, *DP* = 0.457; HM females: *t* = 1.873, *p* = 0.07, *RD* = 23.01 on 30 *df*, *DP* = 0.723; MBM males: *t* = 2.505, *p*<0.05, *RD* = 14.76 on 40 *df*, *DP* = 0.353; HM females: *t* = 1.326, *p*<0.19, *RD* = 26.92 on 40 *df*, *DP* = 0.661). (Figure 3.a.–b.).

## Discussion

In order to investigate whether the weight or the AGD is a suitable indicator of the reproductive capacity in wild mice, we compared a morphological trait (AGD) to litter size and sex ratio in two closely related mice species. We found significant correlation between the AGD and the body weight of female mice at pre-pubertal age in both examined species. In the case of the HM this connection was found in previous studies [35], [45], but no one has yet investigated it in MBM. We also examined the relationship between AGD and body weight of adult mice but it was absent in both species. It can be assumed that in the pre-weaning period the animals have to compete for the limited source of milk, and the pups with larger AGD might be more successful in that as they are more aggressive [30], [50]. It is possible that in a natural environment this difference subsists after weaning but in captivity with ad libitum food availability this phenomenon is blurred.

The overall sex ratio of the litters of HM and MBM mice were 0.49 and 0.51 respectively, which does not differ from the expected 0.5. The sex ratios of litters vary in a wide range in both species. This variability correlates with the mother's AGD in both species. In both HM and MBM females with larger AGD produce male-biased litters; and females with smaller AGD produce female biased. The mother's weight did not correlate with the litter size, neither the litter's sex ratio in any of the investigated species, which is consistent with previous findings by Krackow on wild-caught HM [12].

In some species, connection was found between the mothers' litter size and sex ratio. In litters of ground squirrels, guinea-pigs, wild boars and grass-cutters, positive correlation was found between litter size and the proportion of females. In those species, smaller litters had significantly more males than did large ones [51]–[54]. In the case the investigated species of this study, we did not find any connection between the litter size and litter's sex ratio, however the tendency seems similar. Our not significant results may be caused by the small sample size or the satisfactory diet under laboratory conditions.

The main assumption of the Trivers-Willard hypothesis [1] is that extra parental investment can have a larger effect on the fitness return from the male offspring because males can achieve higher reproductive success than females. Additionally, Williams [3] assumes that in polytocous mammals mothers adjust both their litter size and sex ratio to maximize fitness returns from progeny. Both theories suggest that good maternal condition should result in more sons, and poor condition should result in more daughters. In addition to supporting articles [2], [9], [11], [14], there are a number of recently published articles, which assumes different effects on sex ratio shift in the background. One is the mother's hormonal status at the time of conception: females with high preconception testosterone level tend to produce more male offspring in birds [55], [56] and mammals as well [57]–[61]. Since female mice developing between 2 male siblings in utero have elevated testosterone concentration in their blood at birth [32] and longer AGD [20], [21], [24], IUP may have a long lasting effect on an individual's future reproductive capacity.

In our current study, we failed to provide direct support for the Trivers-Willard/Williams hypothesis because we did not find connection between the mother's weight and their litter's sex ratio or size. However, our results suggest that the mother's intrauterine position is more likely have effect on their litter composition. Our research on two mice species corroborate that prenatal androgenization rather than the mothers condition affect their reproductive traits. A female's AGD is a good predictor of her litter's sex ratio, however a female's AGD depends on their prior IUP. Previous finding in polycoctus mammals shows that the number of male neighbors a female fetus had, affect her litter's sex ratio and litter size in some laboratory species [35], [62]. Same authors' previous report on rabbits showed that the connection between the AGD via the IUP and the sex ratio exist under natural conditions as well [37]. These results confirm that this relationship can be found in wild populations with a much more diverse genetic background than what laboratory strains have.

Although our results did not support the Trivers-Willard-Williams hypothesis, it does not mean it does not exist. A female which develops between two adjacent males in the uterus will have larger AGD. Moreover, it will have higher level of testosterone thus will be more aggressive and likely to become dominant. As the dominant individual it can have access to better resources, better nourishment both quantitatively and qualitatively, which will affect its reproductive success. However in a laboratory environment the ad libitum food may mask this phenomenon.
