# Correction: Anogenital Distance and Condition as Predictors of Litter Sex Ratio in Two Mouse Species: A Study of the House Mouse (*Mus musculus*) and Mound-Building Mouse (*Mus spicilegus*)

**DOI:** 10.1371/annotation/8d7ce17a-7b64-400f-accd-cfdf55d5da3d

**Published:** 2014-01-23

**Authors:** Péter Szenczi, Oxána Bánszegi, Zita Groó, Vilmos Altbäcker

The citation of Reference number 9 is incorrect. The correct citation is: 

Dušek A, Bartoš L, Sedláček F (2011) Mixed sex allocation strategies in a polytocous mammal, the house mouse (Mus musculus). Behav Ecol Sociobiol 65: 2209–2217.

